# Looking forward to volume six

**DOI:** 10.3762/bjoc.6.1

**Published:** 2010-01-06

**Authors:** Jonathan Clayden

**Affiliations:** 1 School of Chemistry, University of Manchester, Oxford Rd., Manchester M13 9PL, UK

January 2010 sees the beginning of the sixth volume of the *Beilstein Journal of Organic Chemistry*. Launched in August 2005, BJOC became the first journal in the field of organic chemistry fully conforming to the open access model, and our first five years have been a time of fast growth and development. Fully supported by the Beilstein-Institut (we have no submission fees and no access charges), the journal continues to publish top quality work in all fields of organic chemistry, from materials, through mechanism, synthesis and natural products, to chemical biology.

In the last few months we have seen a rapid rise in the number of papers we are publishing. For example, 2009 saw an increase in submissions of 50% over the previous year, and the number of papers we have published continues to increase year on year (over 50% more in 2009 than 2008) with no compromise to our original target of publishing the highest quality organic chemistry available freely online. Supporting this increasing publication rate we now have a growing team of associate editors from across Europe and North America (with plans to extend to remaining continents). Every paper is assessed in detail by an editor and thoroughly refereed by an international panel of referees drawn from all fields of organic chemistry. Underpinning this, the whole manuscript management, production and publication system we use was developed specifically for BJOC to be organic chemistry specific, and is supported by a dedicated team at the Beilstein-Institut.

The open access model allows us to be fully available to all users, and the number of people accessing our website and reading our papers is now over 20000 per month, an increase of 50% over the last year. The online-only format makes universal access to all aspects of the journal possible worldwide. It also brings with it other advantages, such as our innovative “album” tool for a quick graphic structural overview of the contents of a paper. A further development in the last two years has been our pioneering of “thematic series”. These are a kind of “virtual special issue” in which experts in a field, brought together under the guest editorship of a leading scientist, report their latest work in a series of related papers. Published over a period of months in the normal way within the journal, these papers can be viewed together using the “thematic series” tool on the website. Recent series have covered the topical themes of flow chemistry [1], supramolecular chemistry [2] and liquid crystals [3], and further series on carbohydrate chemistry and on organofluorine chemistry will appear shortly.

The first five years of BJOC have been an exciting time to be involved in pioneering this project - challenging in many ways, but rewarding as the success and profile of the journal continues to build. As we move into a new decade, it is certain that scientific opinion and publication habits are broadly moving in the direction of Open Access. The Open Access model has come late to chemistry, but is set to become the dominant publishing model of the future and BJOC remains well placed to be in the vanguard of Open Access journals in the field.

Jonathan Clayden

Editor-in-chief

Manchester Dec 2009
